# Yeast Surface-Displayed H5N1 Avian Influenza Vaccines

**DOI:** 10.1155/2016/4131324

**Published:** 2016-12-19

**Authors:** Han Lei, Sha Jin, Erik Karlsson, Stacey Schultz-Cherry, Kaiming Ye

**Affiliations:** ^1^Department of Biomedical Engineering, Watson School of Engineering and Applied Sciences, Binghamton University, State University of New York (SUNY), Binghamton, NY 13902, USA; ^2^Department of Infectious Diseases, St. Jude Children's Research Hospital, Memphis, TN 38105, USA

## Abstract

Highly pathogenic H5N1 avian influenza viruses pose a pandemic threat to human health. A rapid vaccine production against fast outbreak is desired. We report, herein, a paradigm-shift influenza vaccine technology by presenting H5N1 hemagglutinin (HA) to the surface of yeast. We demonstrated, for the first time, that the HA surface-presented yeast can be used as influenza vaccines to elicit both humoral and cell-mediated immunity in mice. The HI titer of antisera reached up to 128 in vaccinated mice. A high level of H5N1 HA-specific IgG1 and IgG2a antibody production was detected after boost immunization. Furthermore, we demonstrated that the yeast surface-displayed HA preserves its antigenic sites. It preferentially binds to both avian- and human-type receptors. In addition, the vaccine exhibited high cross-reactivity to both homologous and heterologous H5N1 viruses. A high level production of anti-HA antibodies was detected in the mice five months after vaccination. Finally, our animal experimental results indicated that the yeast vaccine offered complete protection of mice from lethal H5N1 virus challenge. No severe side effect of yeast vaccines was noted in animal studies. This new technology allows for rapid and large-scale production of influenza vaccines for prepandemic preparation.

## 1. Introduction

The rapid dissemination of highly pathogenic H5N1 avian influenza viruses and evolution of their antigenic diversity pose a pandemic threat to public health. Vaccination remains one of the most effective measures to prevent severe illness and death from pandemic influenza. Currently, two influenza vaccines are available. One is based on chemically inactivated detergent-solubilized virions composed of hemagglutinin (HA) and other viral proteins, whereas the other is a live attenuated influenza virus vaccine [1, 2]. These two vaccines are produced using fertilized chicken eggs as substrates for propagation. Although the egg-based technology has been successful for the last several decades, it has some critical limitations, including the necessity of choosing appropriate strains in advance, a lengthy manufacturing process, and the need for hundreds of millions of fertilized chicken eggs each year. Clearly, the prediction of seasonal influenza strains in advance is not an easy task, making the mid-course adjustment virtually impossible. Moreover, certain influenza viruses do not propagate well in chicken eggs, leading to a longer production time and few doses available for preventing influenza outbreaks within the shortest time. In addition, many individuals are allergic to eggs and thus cannot receive egg produced vaccines.

One possible solution to these problems is exemplified by developing cell-culture vaccines, a shift in technology that will potentially enable faster production. Another approach is to use reverse genetics for rapid development of influenza vaccines. This approach utilizes a WHO-approved Vero cell line to produce reference vaccine viruses using a plasmid-based reverse-genetic system in less than four weeks [3]. Viral proteins such as HA can be produced using insect cell lines for developing viral-protein based vaccines [4]. Adenoviruses encoding HA can be administrated as vaccines to provide protective immunity against influenza. A single injection of adenovirus-based HA vaccines can protect mice from influenza virus infection [5–8]. Although there was adenovirus serotype 5-based vaccines against influenza virus A/PR/8 (H1N1) in phase I clinical trial [8], natural vector-specific immunity of some human populations against adenovirus serotype 5 could potentially reduce vaccine efficiency in the event that global vaccination against highly pathogenic avian influenza (HPAI) is implemented [9]. Alternatively, a wide range of different human and simian adenovirus serotypes are being developed, which will likely negate the issue of preexisting serotype 5-specific immunity [10–14]. DNA vaccines have also been explored [15–18]. Viral DNA coated gold particles can be injected into the skin with a jet of air. However, the efficacy of the DNA vaccines in humans has yet to be proven.

Yeast-based vaccines have recently been explored for immune protection against influenza viruses. One of the advantages of using yeast as vaccines is that yeast can be rapidly engineered to express new antigen targets [19–23]. Yeast-based vaccines do not require the use of an additional adjuvant such as Aluminum to boost immune response [20]. Compared to intracellular expression of viral proteins, the display of viral proteins on cell surface can facilitate their recognition by host immune system, thereby enhancing their capability of eliciting protective immunity in vaccinated hosts. On the other hand, yeast surface display approach has been developed by other and our research groups [24–28]. It has been employed to develop vaccine against fungal infection, detect protein-protein interaction, monitor glucose concentration, drug screening against influenza virus, and map antibody-antigen binding [22, 25, 29–31]. However, a yeast display system has not been fully investigated for its feasibility as vaccines against fetal influenza virus H5N1 infection. We report, herein, for the first time that H5N1 HA displayed on yeast surface can serve as vaccines to prevent influenza infection. Yeast was chosen due to its capability of postprocessing many mammalian proteins including HA and features of immunostimulatory complexes that can effectively activate dendritic cells (DCs) and stimulate cytotoxic T lymphocytes [19]. Recombinant yeast is capable of delivering heterologous antigens into both major histocompatibility complex class I and II pathways and elicit potent cell-mediated immunity [20]. Our experimental results indicate that the yeast vaccine developed in this study triggers host immune response and provides a complete protection of mice from lethal H5N1 virus infection.

## 2. Materials and Methods 

### 2.1. DNA Cloning and Yeast Culture

*Escherichia coli* (*E. coli*) (New England Biolabs, Beverly, MA),* S. cerevisiae* shuttle plasmid pYD1 (Invitrogen, San Diego, CA), and pcDNA3.1/H5N1/HA/optimized plasmid were used to construct an HA surface display vector.* S. cerevisiae* EBY100 (Invitrogen) served as a host cell for HA surface display. The secretion signal and transmembrane region truncated H5N1 HA gene fragment of A/Vietnam/1203/2004 was PCR-amplified and subcloned into pYD1. The resultant shuttle plasmid pYD1-HA was electroporated into* S. cerevisiae* EBY100. Recombinant yeast transformants were plated on selective minimal dextrose plates containing amino acids (0.67% yeast nitrogen base without amino acids (YNB), 2% glucose, 0.01% leucine, 2% agar, and 1 M sorbitol).* Trp* ^+^ transformants were selected after 3 days of growth. The positive colonies were confirmed by DNA sequencing. Recombinant yeast were cultured in YNB-CAA-Glu (0.67% YNB, 0.5 casamino acids, 2% glucose) and induced by 2% galactose for HA production.* S. cerevisiae* EBY100 bearing pYD1 plasmid served as a negative control for all the experiments.

### 2.2. Western Blot Analysis

1 OD_600_ (1 OD_600_ ≈ 10^7^ cells) equivalent recombinant yeasts were collected after induction for Western blot analysis as described elsewhere [32–34]. A monoclonal mouse anti-HA (H5N1 A/Vietnam/1203/2004) antibody NR-2729 (BEI Resources, Manassas, VA) (1 : 500 dilution) and horseradish peroxidase- (HRP-) conjugated goat anti-mouse IgG (1 : 5000 dilution) (Sigma-Aldrich Co., St. Louis, MO) were used as primary and secondary antibodies.

### 2.3. Glycosylation Analysis of Yeast Surface-Displayed HA

Recombinant yeast was cultured in YNB-CAA-Glu at 30°C overnight and then induced in galactose containing YNB-CAA medium at 20°C for 72 h. Cell pellets from 1 OD_600_ were denatured at 100°C for 10 min. 5000 U of PNGase F (New England Labs) was used according to the manufacturer's instruction. The treated samples were then subjected to Western blot analysis.

### 2.4. HA Receptor-Binding Assay

Biotinylated glycans, Neu5Ac(*α*2-3)Gal(*β*1-4)GlcNAc(*β*1-3)Gal(*β*1-4)GlcNAcb-biotin (3SLNLN-b) and Neu5Ac(*α*2-6)Gal(*β*1-4)GlcNAc(*β*1-3)Gal(*β*1-4)GlcNAcb-biotin (6SLNLN-b), were acquired from the Consortium for Functional Glycomics. Twofold serial diluted glycan solutions were applied to streptavidin-coated plates (Pierce, USA) [35, 36]. 256 HA units collected from the vaccines were used in the assay. Anti-HA mouse serum (1 : 500 dilution) and HRP-conjugated goat anti-mouse IgG (1 : 5000 dilution) were applied. Inactivated H5N1 vaccine (BEI Resource) served as a positive control.

### 2.5. Hemagglutination Assay

Twofold serial diluted 1 OD_600_ equivalent EBY100/pYD1-HA were added to 12-well plates, followed by addition of 1% chicken red blood cells. Agglutination was read after incubation. Inactivated H5N1 vaccine with 90 *μ*g/mL of HA protein (NR-12148) (BEI Resource) served as a positive control.

### 2.6. Immunofluorescence Microscopy

Recombinant yeast was collected and subjected to immunofluorescence assay following the procedure described elsewhere [37]. Mouse anti-HA antibodies (1 : 500 dilution) and goat anti-mouse IgG-FITC conjugates (Sigma) (1 : 5000 dilution) were used.

### 2.7. Flow Cytometry

Recombinant yeasts were collected for flow cytometry analysis using a procedure described elsewhere [38, 39] and FACSAria SPD (BD Bioscience, San Jose, CA). Mouse anti-HA (NR-2729) and FITC-conjugated goat anti-mouse IgG antibodies were used.

### 2.8. Vaccine Preparation

Recombinant* S. cerevisiae* EBY100/pYD1-HA strain was cultured to reach a mid-log growth phase and then induced for the HA display. To elevate the HA display on yeast surface, the culture temperature was lowered from 30°C to 20°C after galactose-induction. The cells were collected at 72 h after induction by centrifugation at 3,200 ×g at 4°C for 10 min and then washed with PBS. The cells were heat-inactivated at 60°C for 1 h. The final concentration of the vaccines was adjusted to 1 OD_600_/*μ*L using PBS and stored at 4°C until use.

### 2.9. Vaccination

Six- to eight-week-old female BALB/c mice were acquired from the Jackson Laboratories (Bar Harbor, ME). The mice were housed in the pathogen-free (SPF) University Animal Center. All animal experiments were reviewed and approved by the University and St. Jude Children's Research Hospital IACUCs. Groups of 21 mice each were either intramuscularly (IM) or intraperitoneally (IP) immunized with 50 *μ*L (containing 50 OD_600_ equivalent yeast vaccines) of heat-inactivated EBY100/pYD1-HA vaccines, PBS, EBY100/pYD1 (a mock plasmid transformed yeast), and 3 *μ*g HA containing inactivated H5N1 vaccines, respectively. These mice received a boost immunization two weeks later. Sera were collected at day 13, day 28, and five months after prime immunization and tested for HA antibody production.

### 2.10. Influenza Virus Challenge

Challenge studies were performed under biosafety level 3+ containment at the St. Jude Children's Research Hospital. Briefly, groups of 10 vaccinated mice each were anesthetized and intranasally challenged with 25 *μ*L 10 × 50% lethal dose (LD_50_) A/Vietnam/1203/2004 H5N1 virus at day 14 after boost. The lung virus titer was detected at day 3 postinfection. Clinical signs and body weight were monitored twice daily for 14 days after virus challenge. Clinical scores (0–4) were determined as follows: 0 = no clinical signs; 1 = rough coat; 2 = rough coat, less reactive, and passive during handling; 3 = rough coat, rolled up, labored breathing, and passive during handling; and 4 = rough coat, rolled up, labored breathing, and unresponsive. Animals with a score of 4 were euthanized.

### 2.11. Characterization of Immune Response

HA-specific antibody responses were determined using a standard enzyme-linked immunosorbent assay (ELISA) with avidin-biotin system [32]. H5N1 A virus A/Vietnam/1203/2004 recombinant HA (BEI Resource) at a concentration of 2 *μ*g/mL was used. Biotinylated goat anti-mouse IgG, IgG1, and IgG2 (1 : 5000 dilution) (R&D Systems, Minneapolis, MN), 1.25 mM pNPP phosphatase substrate (MP Biomedicals, Santa Ana, CA), and 2.21 mM hydrogen peroxide (Millipore, Temecula, CA) were used.

IFN-*γ* and IL-4 secreting cells were determined using an ELISpot kit (R&D Systems). Goat anti-mouse IFN-*γ* and IL-4 antibodies were used (Millipore, USA). Splenocytes (1 × 10^6^ cells/well) that were freshly prepared from vaccinated mice were seeded to the plates and stimulated with HA-specific peptides (ISVGTSTLNQRLVP) (BEI Resources). This peptide was derived from A/Vietnam/1203/2004 H5N1 HA. The plates were treated sequentially with biotinylated goat anti-mouse IFN-*γ* (1 : 5000 dilution) and IL-4 antibodies (1 : 5000 dilution), alkaline phosphatase conjugated streptavidin (1 : 1000 dilution), and the substrate solution (BCIP/NBT Chromogen) to reveal the spots. The spots were analyzed using ELISpot (CTL ImmunoSpot Analyzer, OH, USA).

### 2.12. Hemagglutination Inhibition (HI) Assay

Immune serum neutralization activity was determined by HI assay. Sera collected from vaccinated mice were treated with a receptor destroying enzyme from* Vibrio cholera* (Denka-Seiken, San Francisco, CA) before testing for the presence of H5 specific antibodies, as reported previously [40]. Sera were mixed with 4 HA units of inactivated A/Vietnam/1203/2004 vaccine (BEI Resource) and incubated with 0.5% (v/v) chicken red blood cells.

### 2.13. Statistical Analysis

Statistical analyses of the experimental data were performed by one-way factorial analysis of variance. A *p* value < 0.05 was considered as a statistical significance. All experiments were carried out in triplicate.

## 3. Results and Discussion

### 3.1. Functional Display of Glycosylated HA on Yeast Surface

**Figure 1:**
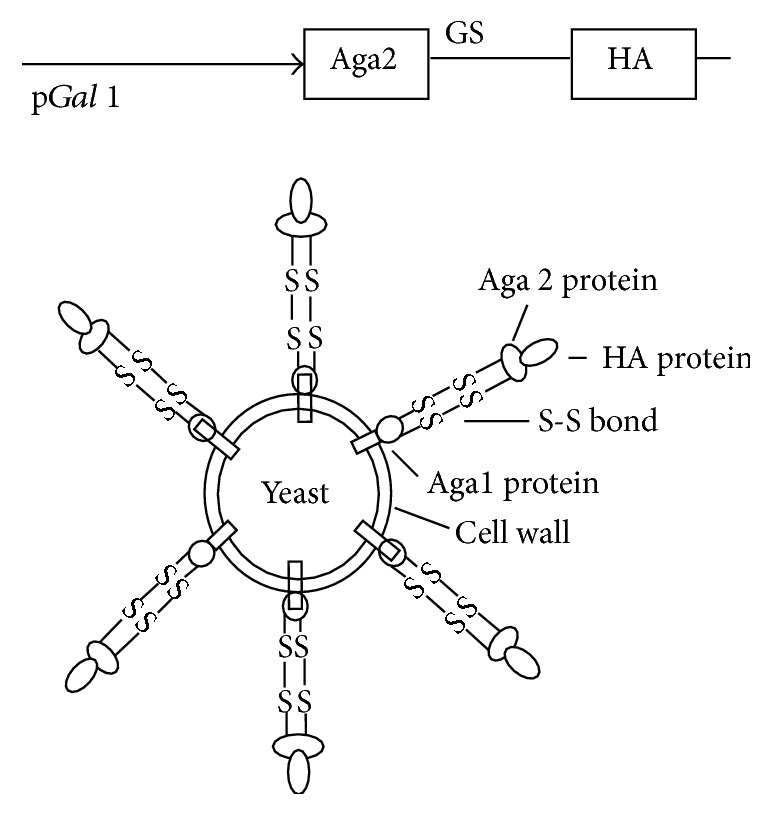
Functional display of HA on yeast surface. A schematic diagram of HA display on yeast surface. The Aga2-HA fusion protein binds to Aga1 through two disulfide bonds after its secretion from the yeast. Aga1 is the first subunit of the yeast a-agglutinin receptor. It is secreted from the cell and becomes covalently attached to *β*-glucan in the extracellular matrix of the yeast cell wall. A GS linker is inserted between Aga2 and HA to stabilize the fusion protein expression.

**Figure 2:**
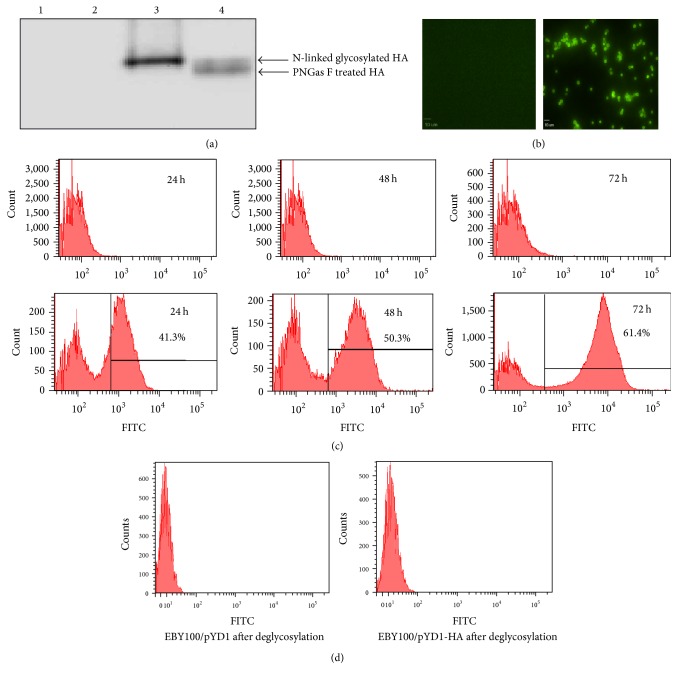
HA displayed on yeast surface. (a) Western blot of yeast surface-displayed HA. Lane 1: EBY100/pYD1 transformed yeast, Lane 2: PNGase F treated EBY100/pYD1 transformed yeast, Lane 3: EBY100/pYD1-HA transformed yeast, and Lane 4: PNGase F treated EBY100/pYD1-HA transformed yeast. (b) Immunofluorescence microscopy of EBY100/pYD1 (left) and EBY100/pYD1-HA (right) yeast at 72 h after induction. Scale bar: 10 *μ*m. Yeast surface-displayed HA was immunostained with mouse anti-HA antibodies and goat anti-mouse IgG-FITC conjugates antibodies. (c) Flow cytometric analysis of HA displayed on yeast surface at 24, 48, and 72 h after induction. Top panel: EBY100/pYD1; bottom panel: EBY100/pYD1-HA. Yeast surface-displayed HA was immunostained with mouse anti-HA antibodies and goat anti-mouse IgG-FITC conjugates antibodies. (d) Flow cytometric analyses of EBY100/pYD1 and EBY100/pYD1-HA after deglycosylation.

The subcloning of A/Vietnam/1203/2004 H5N1 truncated HA gene into a yeast surface protein display vector pYD1 led to the fusion of HA to C-terminus of Aga2, the second subunit of the yeast a-agglutinin receptor (Figure 1). The N-terminus of Aga2 entails both a secretion signal peptide and a binding site for Aga1, another subunit of the yeast a-agglutinin. The Aga2-HA was expressed and bound to Aga1 through two disulfide bonds, resulting in the HA display on yeast wall. Mammalian glycoproteins can be glycosylated in* S. cerevisiae *[41]. Nonetheless, yeast synthesizes only high mannose-type sugars, leading to the production of N-linked glycosyl chains similar to the mannose-rich core oligosaccharides attached to the asparagine residues in mammalian glycoproteins. Our experimental result showed that N-linked glycosylation of HA occurs on the yeast surface (Figure 2(a)). The treatment of cell-free extracted HA with endoglycosidase PNGase F that removes N-linked glycosyl chains from a peptide backbone revealed two distinct bands in Figure 2(a). HA was displayed on yeast surface as early as 24 h after induction (Figure 2(b)). HA surface-displayed yeast increased over time, reaching approximately 61% at 72 h after induction (Figure 2(c)). Furthermore, no fluorescence signal could be detected after treating EBY100/pYD1-HA with endoglycosidase PNGase F (Figure 2(d)), whereas non-PNGase F treated EBY100/PYD1-HA exhibited strong fluorescence signals, indicting the binding of FITC labeled anti-HA antibodies to yeast surface-displayed HA. These results demonstrated the completion of surface glycosylation of HA, which is a critical posttranslation modification for immunogenicity of the vaccines.

### 3.2. Yeast Surface-Displayed HA Preserves Its Antigenic Sites

**Figure 3:**
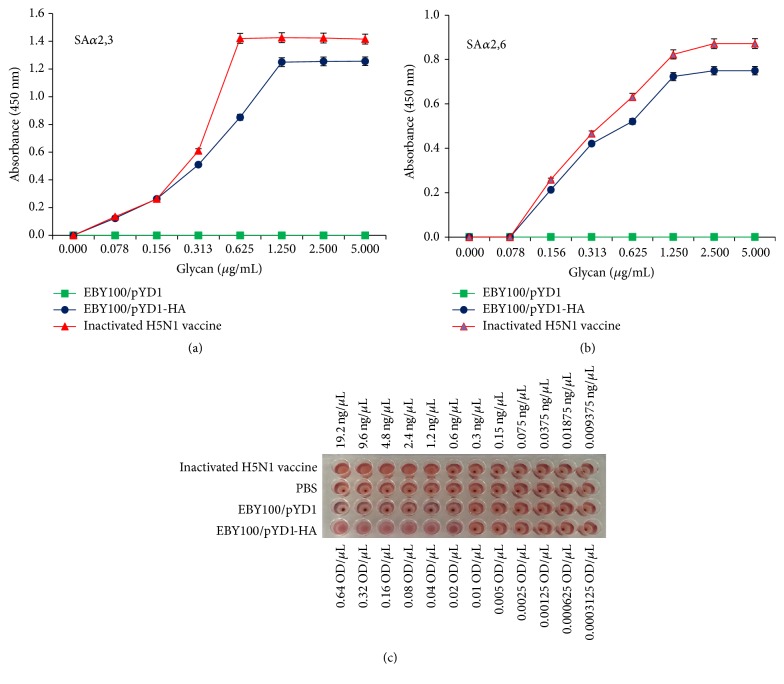
Receptor-binding specificity of yeast surface-displayed HA. Yeast surface-displayed HA binds to (a) *α*2,3-linked sialylglycan receptor (SA*α*2,3) and (b) *α*2,6-linked sialylglycan receptor (SA*α*2,6). EBY100/pYD1 served as a negative control, and an inactivated H5N1 vaccine as a positive control. Error bars represent standard deviations of the mean, which is calculated with three independent repeats. (c) Results of hemagglutination assay. Inactivated H5N1 vaccine served as a positive control, and PBS and EBY100/pYD1 as two negative controls.

Two biotinylated glycans, Neu5Ac(*α*2-3)Gal(*β*1-4)GlcNAc(*β*1-3)Gal(*β*1-4)GlcNAcb-biotin (SA*α*2.3) and Neu5Ac(*α*2-6)Gal(*β*1-4)GlcNAc(*β*1-3)Gal(*β*1-4)GlcNAcb-biotin (SA*α*2.6), were used for interrogating whether the yeast surface-displayed HA preserves its antigenic sites. SA*α*2.3 and SA*α*2.6 are avian-type and human-type receptor for influenza HA, respectively. With the disulfide bond breakage by DTT between Aga1-Aga2-HA fusion protein, the glycan binding specificity of these isolated HAs was characterized (Figures 3(a) and 3(b)). Yeast surface-displayed HA preferentially binds to SA*α*2,3, similar to that observed with inactivated H5N1 vaccines. It also binds to SA*α*2,6. In contrast, yeast that did not display HA on their surface cannot bind to either type of glycan. HA of A/Vietnam/1203/2004 (H5N1) was isolated from a human transmittable avian influenza strain. It exhibits a high binding affinity for both avian and human receptors. These characterizations suggested that yeast HA vaccines developed in this study are able to bind to both avian- and human-type receptors. To quantify HA displayed on the yeast surface, we determined hemagglutination activity of the vaccine using inactivated H5N1 vaccine with known HA content (Figure 3(c)). We estimated that 1 OD_600_ yeast vaccines contain 60 ng of HA. This assay helped determine the doses used for yeast vaccine immunization.

### 3.3. Yeast Surface-Displayed HA Elicits Humoral Immune Responses in Animals

**Figure 4:**
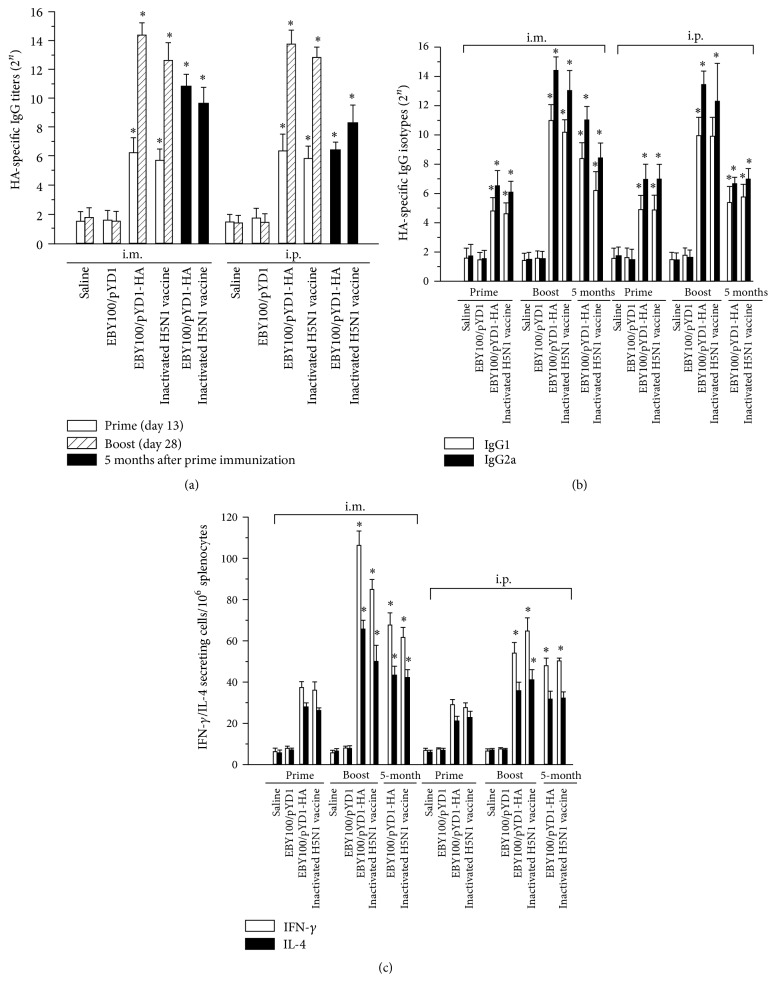
Detection of humoral and cellular immune response elicited by HA surface-displayed vaccines in immunized mice. Sera were collected from vaccinated mice and tested for production of (a) HA-specific IgG antibodies at day 13 (*n* = 21 mice/group), day 28 (*n* = 21 mice/group), and 5 months (*n* = 5 mice/group) after prime immunization and (b) IgG isotypes. (c) The numbers of IFN-*γ*/IL-4 secreting T cells in vaccinated mice. Splenocytes were isolated from vaccinated mice at day 10 (*n* = 3 mice/group) and day 24 (*n* = 3 mice/group) after the prime immunization and evaluated by ELISpot. Data are represented as mean ± SD. Asterisk indicates significant difference, as compared to the controls (PBS and EBY100/pYD1 injected mice) (^*∗*^ *p* < 0.05).

To investigate whether the HA-presenting yeast can serve as vaccines to elicit a protective immune response, the recombinant yeast was tested in an animal model. Four groups of 16 mice each were immunized with IM or IP injection of 50 OD_600_ equivalent heat-killed EBY/pYD1-HA yeast vaccines in 50 *μ*L of 0.9% saline and then boosted at day 14 after prime immunization. EBY/pYD1 and 0.9% saline served as negative controls, whereas inactivated H5N1 vaccines as a positive control. Weight and activity of immunized mice were monitored closely to examine any adverse impact on mice. No significant weight loss or reduction in activity was observed in immunized mice. Sera collected at days 13, 28, and 150 after prime vaccination were analyzed for anti-HA IgG production. As indicated in Figure 4(a), an elevated production of anti-HA antibodies was observed in EBY100/pYD1-HA vaccinated mice but not in negative control group of mice. We observed a 512-fold increase in anti-HA antibody production after the boost immunization in EBY100/pYD1-HA vaccinated mice (Figure 4(a)). HA-specific antibody production level elicited by the vaccines was slightly higher than that induced by H5N1 vaccines. The production of HA-specific IgG antibodies in EBY100/pYD1-HA vaccinated mice could be detected five months after vaccination.

### 3.4. HI Titers and Cross-Reactivity of Yeast Vaccines against Divergent H5N1 Viruses

The HI titration was performed to further confirm the immunity of the yeast vaccine. The HI titer of sera collected from EBY100/pYD1-HA vaccinated mice after boost increased up to 128 (Table 1). Serum HI antibody titer of higher than 40 is considered sufficient for protecting from influenza virus infection. Compared to IM vaccine administration, the HI titer of anti-HA antibody produced in IP administrated mice was much lower, suggesting that IP administration is not as efficient as IM administration. Furthermore, we observed continuous production of anti-HA antibodies for more than five months in IM vaccinated mice. To determine whether the yeast vaccines can provide protection against other H5N1 viruses, we performed HI assay using sera collected from yeast vaccinated mice against A/Vietnam/1203/2004 (H5N1), A/duck/Laos/3295/2006 (H5N1), and A/duck/Hunan/795/2002 (H5N1). Sera collected from the inactivated H5N1 vaccinated mice served as a control for these analyses. Inactivated H5N1 vaccines were administrated by following the same prime/boost immunization schedule used for yeast vaccines. As shown in Table 1, yeast vaccines exhibited a higher cross-reactivity to both homologous and heterologous H5N1 viruses when administrated intramuscularly. The intramuscular administrated inactivated H5N1 vaccines showed a high cross-reactivity to homologous but not to heterologous H5N1 viruses.

### 3.5. Yeast Surface-Displayed HA Induces Both IgG1 and IgG2a Expression in Vaccinated Mice

BALB/c mice typically respond to inactivated influenza vaccines and subunit vaccines with a Th2-type immune response, which is associated with the stimulation of IgG1 antibodies [42]. However, a significant production of IgG2a has been observed in the sera of mice that survive viral infections [43]. The IgG2a is stimulated during a Th1-type immune response. Stimulation of IgG2a antibodies is associated with increased efficacy of influenza vaccination [43, 44]. We observed that HA-specific IgG1 and IgG2a expression levels increased considerably in EBY100/pYD1-HA vaccinated mice after boost (Figure 4(b)). The expression level of IgG2a was higher than that of IgG1 in vaccinated mice. Induction of both IgG1 and IgG2a elevates vaccine efficacy and helps improve the efficiency of virus clearance after infection [43].

### 3.6. Yeast Displayed HA Elicits Cell-Mediated Immune Response in Vaccinated Mice

Furthermore, we determined whether the yeast vaccines induced a T cell-mediated immune response. Splenocytes were harvested at day 10 after prime and boost immunization, respectively. We observed a significant increase in both interferon-*γ* (IFN-*γ*) and interleukin-4 (IL-4) production in vaccinated mice after boost (Figure 4(c)), suggesting the induction of an adaptive immune response in these vaccinated mice. A considerably higher level IFN-*γ* production, compared to the IL-4 production, suggested a Th-1 biased cellular immune response elicited by vaccines in immunized mice, which plays a critical role in preventing virus infection [45, 46]. Results of low levels of cellular immune responses in IP vaccinated mice are consistent with low HI titers of antisera detected in these mice.

### 3.7. Yeast Surface-Displayed HA Vaccines Offer Complete Protection from Influenza Infection

**Figure 5:**
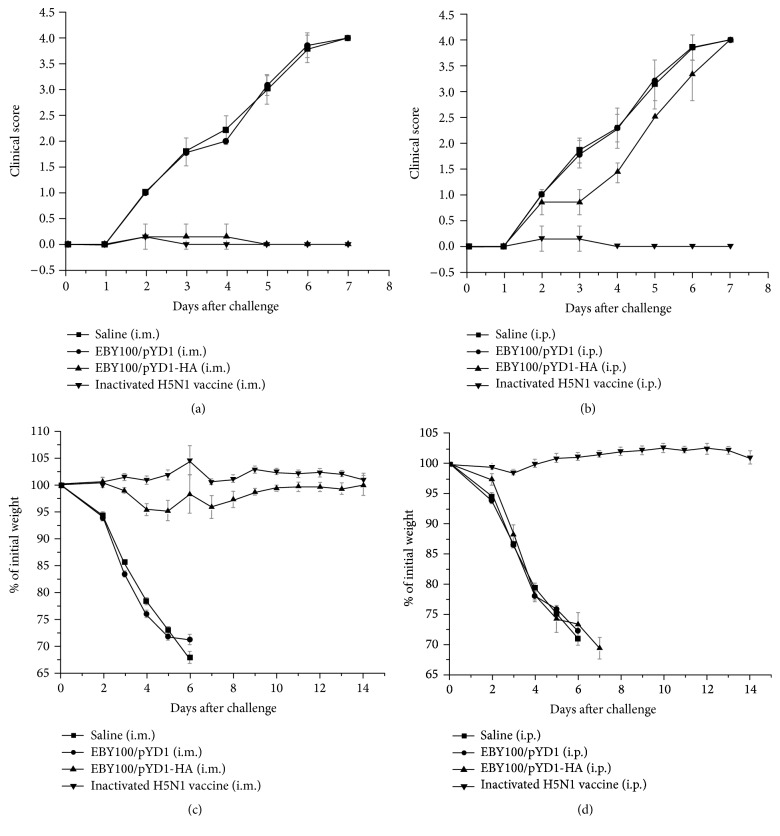
Immune protection conferred by EBY100/pYD1-HA against lethal challenge with homologous H5N1 virus. (a-b) Clinical score on the intramuscularly (i.m.) or intraperitoneally (i.p.) vaccinated mice at 7 days after challenge and (c-d) weight change as a percentage on the intramuscularly (i.m.) or intraperitoneally (i.p.) vaccinated mice after challenge.

**Figure 6:**
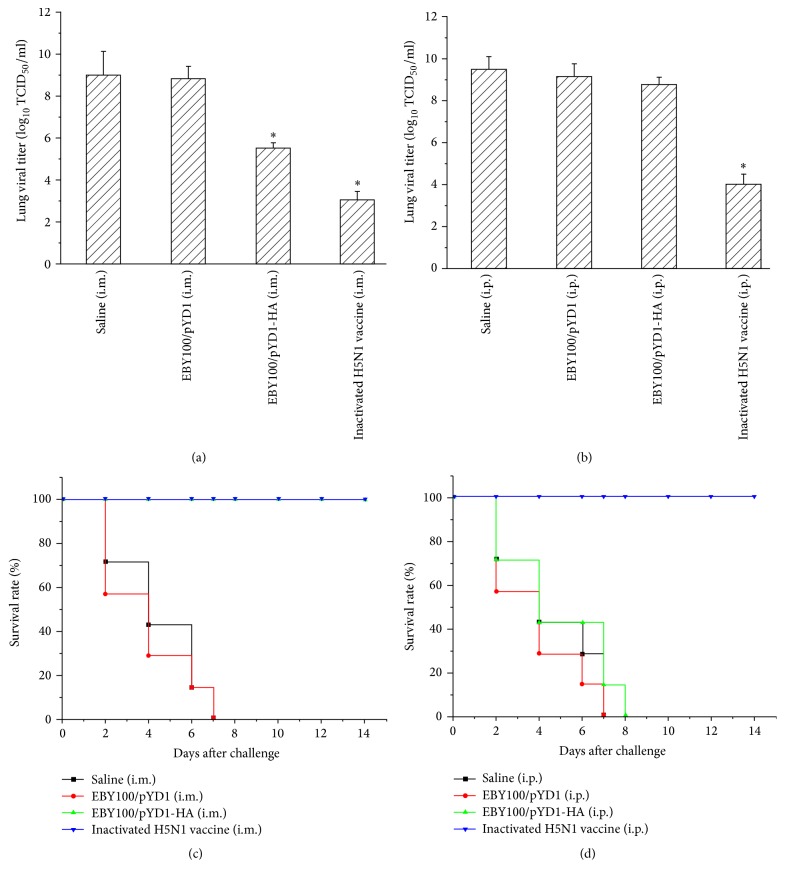
Viral titers and survival rate in vaccinated mice after lethal challenge with homologous H5N1 virus. (a-b) Lung viral titers determined at day 3 after virus challenge (*n* = 3 of 10 challenged mice). PBS and EBY100/pYD1 injected mice were used as controls. ^*∗*^ *p* < 0.05. (c-d) Survival rate of mice after virus challenge (*n* = 7 mice/group). Inactivated H5N1 vaccine administrated mice served as positive controls.

In addition, we determined protection of mice from lethal avian influenza H5N1 infection through HA-presented yeast vaccination. Mice (*n* = 10) were challenged with 25 *μ*L 10 × LD_50_ of H5N1 A/Vietnam/1203/2004 virus at two weeks after boost and observed for 14 days after challenge. Inactivated H5N1 influenza vaccines served as a positive control. As shown in Figure 5, mice in negative control groups (Saline and EBY100/pYD1) showed clinical symptoms of severe disease (Figures 5(a) and 5(b)), including significant morbidity (as measured by weight loss) at day 5 and mortality by 7 days after challenge (Figures 5(c) and 5(d)) and high lung viral titers (Figures 6(a) and 6(b)). In contrast, IM administrated mice survived and recovered completely in two weeks after challenge (Figure 6(c)). The lung viral titer detected at day 3 after virus challenge dropped significantly in IM vaccine administrated mice (Figure 6(a)), although it was higher than that detected in inactivated H5N1 vaccinated mice. This may suggest that the dose of yeast vaccines needs to be further optimized. Yeast is around 10 *μ*m, making them difficult to be absorbed completely by muscles when administrated IM due to its large size. Oral delivery of these yeast vaccines may circumvent this issue. However, the stability and immunogenicity of these orally delivered yeast vaccines in the gastrointestinal tract need to be carefully examined. No significant weight loss or clinical signs in these mice were noted after virus challenge.

It is noticed that the lung virus titer in IP immunized mice showed a similar level as that observed in the control group, indicating that no protection was induced in these mice. These results are consistent with the virus challenging results. The IP administrated mice had to be humanely euthanized or succumbed to infection at day 8 after challenge. This is consistent with the low HI titer of antisera detected from IP administrated mice and illustrates that the yeast vaccine elicits a better immune response when they are administrated* via* the IM route in mice. Here, we only determined the viral titers in lung of virus challenged mice, as the virus dissemination in the lung is critical to survival of the mice. It should be pointed out that the virus dissemination in other organs such as brain and spleen might also need to be assessed in order to determine whether the yeast vaccine limited extrapulmonary viral dissemination.

It is worth noting that ELISA assay suggested a similar level of antibody response in both IP and IM immunized mice. However, the HI titer of sera collected from IM immunized mice is much higher than that detected from IP immunized mice. As shown in Table 1, the HI titer of sera collected from IM immunized mice reached up to 128 after the second boost, whereas it was 32 in IP immunized mice. It is well documented that serum HI antibody titer of higher than 40 is considered sufficient for protecting (in human) from influenza virus infection, suggesting that IP administration of yeast vaccines is not as efficient as IM immunization. The inconsistence of assay results between ELISA and HI assay might be due to difference in principles between two assays. As we know, the ELISA detects the overall antibody response to HA, whereas the HI measures influenza virus-specific serum antibodies; that is, the antibodies bind to HA receptor-binding site and inhibit virus agglutination of red blood cells. Accordingly, the HI assay is a standard method for determining immunogenicity of a flu vaccine. These discordant results were also reported by Blanchfield et al. [47]. One possibility is that the antibodies produced in IP immunized mice target nonneutralizing regions of HA or they bind with a low affinity, leading to no protection from influenza infection. The lower level of cellular immune response detected in these mice also suggested that yeast vaccines are less effective when they are administrated* via* the IP route in mice.

Taken together, we demonstrated, for the first time, that the yeast vaccine can protect mice from the lethal H5N1 influenza virus infection. The use of mice for evaluating vaccines against highly pathogenic avian influenza viruses has been well documented [5, 48, 49]. We detected prolonged production of antibodies in vaccinated mice. The production of a high level IgG was detected five months after immunization. A higher than 40 of HI titer was detected in sera collected in IM immunized mice after five months, suggesting that yeast vaccine can provide a long-term immunity against influenza virus infection. While five months is an endpoint of our experiment for determining the long-term immunity of the yeast vaccine, it is predicted that the immunity could still be maintained at a higher level after five months. The virus-like particle (VLP) vaccine has been shown to induce long-term protection of mice from influenza virus infection [50, 51]. However, construction and production of these VLP vaccines at an industry scale are cumbersome [52]. Unlike VLP vaccines, yeast vaccines can be produced at large-scale and in a very cost-effective way. In addition, yeast vaccines are safer for use in humans due to the edible nature of* S*.* cerevisiae*, although certain individuals might be allergic to yeast.

The yeast surface protein expression vector system has been well developed by other groups including us [24–26]. We chose* S. cerevisiae rather than P. pastoris *for developing yeast vaccines due to the following considerations.* P. pastoris* is a more attractive yeast alterative for recombinant protein expression due to its high yield and high protein expression efficiency. Nonetheless, it is not a good host for HA display. Our group (data not shown) and others such as Wasilenko et al. [53] and Boder and Wittrup [26] suggested that the surface HA display is less efficient in* P. pastoris.* Furthermore,* S. cerevisiae*-based yeast vaccines can potentially be used as oral vaccines owing to their edibility. The oral vaccination could elicit both humoral and cellular immune response at systematic and mucosal sites [54]. However, their stability and immunogenicity need to be carefully characterized when delivered to the gastrointestinal tract.

## 4. Conclusions

We developed a new type of avian influenza vaccines by presenting H5N1 HA to the surface of yeast. Our experimental results demonstrated that the HA surface-displayed recombinant yeast vaccines elicited not only humoral but also cell-mediated immunity in mice, providing a complete protection of mice from lethal H5N1 virus infection. No severe side effect was observed in vaccinated mice, suggesting its potential safe use in humans. These yeast vaccines can be fermented at a low cost, allowing for rapid and large-scale production of influenza vaccine for preventing influenza outbreaks.

## Figures and Tables

**Table 1:** Cross-reactivity of yeast vaccine immunized mice sera against divergent H5N1 viruses.

	HI Titer								
	VN/1203/04 H5N1			A/duck/Laos/3295/2006 H5N1			A/duck/Human/795/2002 H5N1		
	Prime	Boost	5 months	Prime	Boost	5 months	Prime	Boost	5 months
i.m.									
Saline	2	4		4	4		2	4	
EBY100/pYD1	2	4		4	4		4	4	
EBY100/pYD1-HA	25.4	128^*∗*^	40.3^*∗*^	16	40.3^*∗*^	32	16	40.3^*∗*^	32
Inactivated H5N1	32	128^*∗*^	40.3^*∗*^	25.4	32	32	25.4	32	16
i.p.									
Saline	2	4		2	4		4	4	
EBY100/pYD1	4	4		4	8		2	4	
EBY100/pYD1-HA	16	32	25.4	16	32	16	16	16	16
Inactivated H5N1	40.3	128^*∗*^	40.3^*∗*^	32	32	16	25.4	32	25.4

*Note*: HI titers are presented as geometric means. Asterisks indicate statistically significant *p* < 0.05 as compared with that detected in saline and EBY100/pYD1 treated mice (*n* = 21 per group).
